# A neonate with multiple hand flexor tendon ruptures due to methicillin-susceptible *Staphylococcus aureus* sepsis: a case report

**DOI:** 10.1186/s12887-023-03871-z

**Published:** 2023-02-09

**Authors:** Tomomi Nakamuara, Masanori Iwai, Takeshi Inoue, Hiroki Irie, Tatsuki Karasugi, Atsuhito Seki, Masayoshi Hamaguchi, Shohei Kuraoka, Tomoyuki Mizukami, Kimitoshi Nakamura

**Affiliations:** 1 Department of Pediatrics, Kumamoto University Hospital, 1-1-1 Honjo, 860-8556 Chuoku, Kumamoto, Japan; 2 Department of Emergency Medicine and Critical Care, Kumamoto University Hospital, 1-1-1 Honjo, 860-8556 Chuoku, Kumamoto, Japan; 3 Department of Orthopedic Surgery, Kumamoto University Hospital, 1-1-1 Honjo, 860-8556 Chuoku, Kumamoto, Japan; 4 Department of Orthopedic Surgery, National Center for Child Health and Development, 2-10-1 Ohkura, 157-8535 Setagayaku, Tokyo, Japan; 5 Department of Pediatrics, National Hospital Organization Kumamoto Medical Center, 1-5 Ninomaru, 860-0008 Chuoku, Kumamoto, Japan

**Keywords:** Neonate, Pyogenic tenosynovitis, Tendon rupture, Ultrasonography, Case report

## Abstract

**Background:**

Neonatal pyogenic tenosynovitis is a highly emergent soft tissue infection. We report a case of a neonate with pyogenic tendinopathy and tendon rupture diagnosed by ultrasonography (US). He subsequently developed pyogenic arthritis and osteomyelitis during antimicrobial therapy.

**Case presentation:**

A 7-day-old boy was admitted to our hospital with redness and swelling of the right index finger. US on admission showed rupture of the flexor tendon of the right index finger with inactivity. The day after admission, he developed pyogenic arthritis of the right elbow and, subsequently, pyogenic osteomyelitis. *Staphylococcus aureus* was identified through bacterial culture, and the patient was treated with intravenous antibiotics for 6 weeks. However, after discharge from our hospital, rupture of the flexor tendon of the left thumb was confirmed. A two-stage flexor tendinoplasty was completed at the age of 2 years and 1 month for the flexor tendon rupture on his right index finger.

**Conclusions:**

In addition to blood culture, ultrasonographic evaluation should be performed in neonates with erythematous and swollen joints to identify the focus of infection as soon as possible. Moreover, repeated regular US examination is important in the follow-up of bone and soft tissue infections.

## Background

Pyogenic tenosynovitis is caused by bacterial infection of the synovial sheath, resulting in finger dysfunction and, in severe cases, amputation [1, 2]. Because there are no detailed reports on pyogenic tenosynovitis in neonates, the pathogenesis, diagnostic method, progression, and prognosis of the disease in these patients remain unclear. We treated a neonate who developed multiple pyogenic tenosynovitis caused by methicillin-susceptible *Staphylococcus aureus* (MSSA), thought to have invaded through the needle puncture used for infusion. In this case, ultrasonography (US) was essential to diagnose pyogenic tenosynovitis. Furthermore, late-onset osteomyelitis of the proximal end of the right radius and ulna was detected by weekly radiography. After antibiotic treatment, flexor tendon rupture was found on another finger during follow-up US. A two-stage flexor tendinoplasty was completed at the age of 2 years and 1 month. We discuss the case of pyogenic tenosynovitis in a neonate with onset on postnatal day 7. This report details the diagnostic examination and treatment of the patient, including plastic surgery.

## Case presentation

### Patient

A 7-day-old boy.

### Symptoms

Erythema and swelling around the proximal interphalangeal (PIP) joint of the right index finger.

### Perinatal history

The mother had her first pregnancy through ovulation induction. She delivered diamniotic dichorionic twin boys at 36 weeks and 6 days by scheduled cesarean section. He was the second child of twins.

### Family history

The patient has no family history of immunodeficiency, such as chronic granulomatosis or neonatal early-onset bacterial infection. The family history accounts for his twin brother as well.

### Current medical history

The patient was admitted to the neonatal intensive care unit (NICU) of previous doctor because he was a preterm infant with low birth weight (2,012 g) and hypoglycemia (42 mg/dL). A doctor wearing non-sterile gloves secured the infusion route for 10% dextrose at the dorsal right hand after wiping with an alcohol swab. A sterilized film protected the insertion site, which was non-erythematous upon observation, and was performed every 8 h. Oral feeding was started at 10 h of age, and fluid infusion was gradually reduced. On postnatal day 3, non-erythematous swelling was noted at the infusion site, and the needle was removed and wiped with alcohol. The patient deteriorated on postnatal day 6. The following day, his right index finger was erythematous and swollen. The patient also became apneic. After collecting blood cultures, sepsis and bacterial arthritis were considered, and the patient was transported to our NICU for orthopedic surgery therapy.

### Condition on admission

**Fig. 1:**
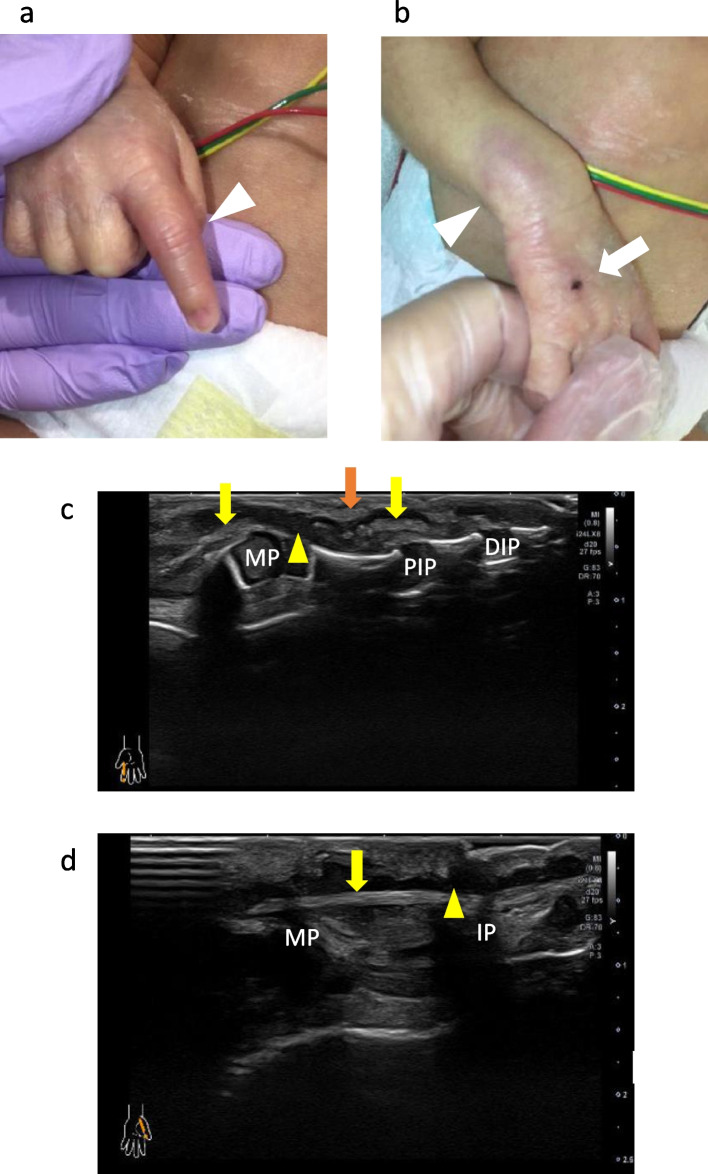
**a** Redness and swelling of the right index finger’s proximal interphalangeal (PIP) joint are observed (arrowhead). The PIP joint presents impairment in spontaneous flexion. **b** Scab formation and redness are observed around the indwelling needle removal mark on the dorsum of the right hand (arrow), and purpura is seen on the wrist (arrowhead). **c** Ultrasound imaging of the right index finger on the day of hospitalization. The flexor digitorum profundus tendon (yellow arrows) is torn and contracted, and the flexor digitorum superficialis tendon (orange arrow) is torn and relaxed. Fluid accumulation is seen in the tendon sheath (yellow arrowhead). **d** The flexor pollicis longus tendon is intact (yellow arrow), but fluid accumulation is seen in the tendon sheath (yellow arrowhead)

Body temperature, 38.5 °C; heart rate 186/min, respiratory rate 52/min, blood pressure 72/51 mmHg, SpO_2_ 100% (FiO_2_ 0.21). The right index finger was red and swollen, and the distal interphalangeal (DIP) and PIP joints were extended with no spontaneous flexion (Fig. 1a, arrowhead). Scab formation and peripheral erythema were observed around the indwelling needle removal mark on the dorsomedial aspect of the right hand (Fig. 1b, arrow), and purpura was observed on the wrist (Fig. 1b, arrowhead). Redness was also observed around the interphalangeal (IP) joint of the left thumb and the sacral region. His laboratory data are shown in Table 1.

**Table 1:** Blood tests on admission

WBC	12300	/μL	CRP	15.86	mg/dL	PT	15	%
Stab	7.0	%	PCT	0.95	ng/μl	PT(INR)	3.9	
Seg	56.0	%				APTT	23	%
Lymph	23.0	%	IgG	499	mg/dL	Fib	361	mg/dL
Mono	11.0	%	IgA	5	mg/dL	FDP	16.4	μg/mL
Eosin	3.0	%	IgM	113	mg/dL	D-dimer	6.2	μg/mL
RBC	440 × 10^4^	/μL						
Hgb	16.7	g/dL	C3	127.7	mg/dL	AST	20	U/L
Hct	44.5	%	C4	17.7	mg/dL	ALT	6	U/L
Plat	6.5 × 10^4^	/μL	CH50	< 10	U/mL	LDH	373	U/L

FDP was 3.2 times higher than our hospital standard. He was diagnosed with overt disseminated intravascular coagulation (DIC) based on Japanese diagnostic criteria (platelet ≤ 70,000/μL, PT-INR ≥ 1.8, FDP ≥ 2.5 times the institutional standard).

### US examination

The right index finger revealed a flexor digitorum profundus (FDP) tendon rupture (Fig. 1c, yellow arrows), a flexor digitorum superficialis (FDS) tendon rupture (Fig. 1c, orange arrow), and a hypoechoic area (Fig. 1c yellow arrowhead) in the tendon sheath from the metacarpophalangeal (MP) joint to the PIP joint. The continuity of the flexor pollicis longus (FPL) tendon in the left thumb was preserved (Fig. 1d, yellow arrow), but a hypoechoic area (Fig. 1d, yellow arrowhead) was found within the tendon sheath and was thought to be fluid accumulation. The next day, redness and swelling of the right elbow joint were noted. US showed hyperechoic fluid accumulation and floaters in the right elbow joint capsule, and a sub-echo puncture collected 5 mL of pus. Radiographic examination showed no abnormal findings, and magnetic resonance imaging (MRI) revealed an abnormal signal under the skin of the right upper arm, but the subject area was small and difficult to evaluate in detail.

### Treatment course

Meropenem (MEPM; 60 mg/kg/day) and vancomycin (VCM; 40 mg/kg/day) were administered for 10 consecutive days to cover for suspected methicillin-susceptible *Staphylococcus aureus*. Final blood culture reports showed MSSA, and antibiotics were consequently changed to flomoxef (FMOX; 130 mg/kg/day). C-reactive protein (CRP) became negative on the 17th day of admission. FMOX was administered for 32 consecutive days because weekly radiographic examinations showed findings suggestive of osteomyelitis, such as irregular shadows at the proximal ends of the right radius and ulna on the 28^th^ day of admission. Oral cefaclor (40 mg/kg/day) was subsequently administered for a total of 4 weeks.

**Fig. 2:**
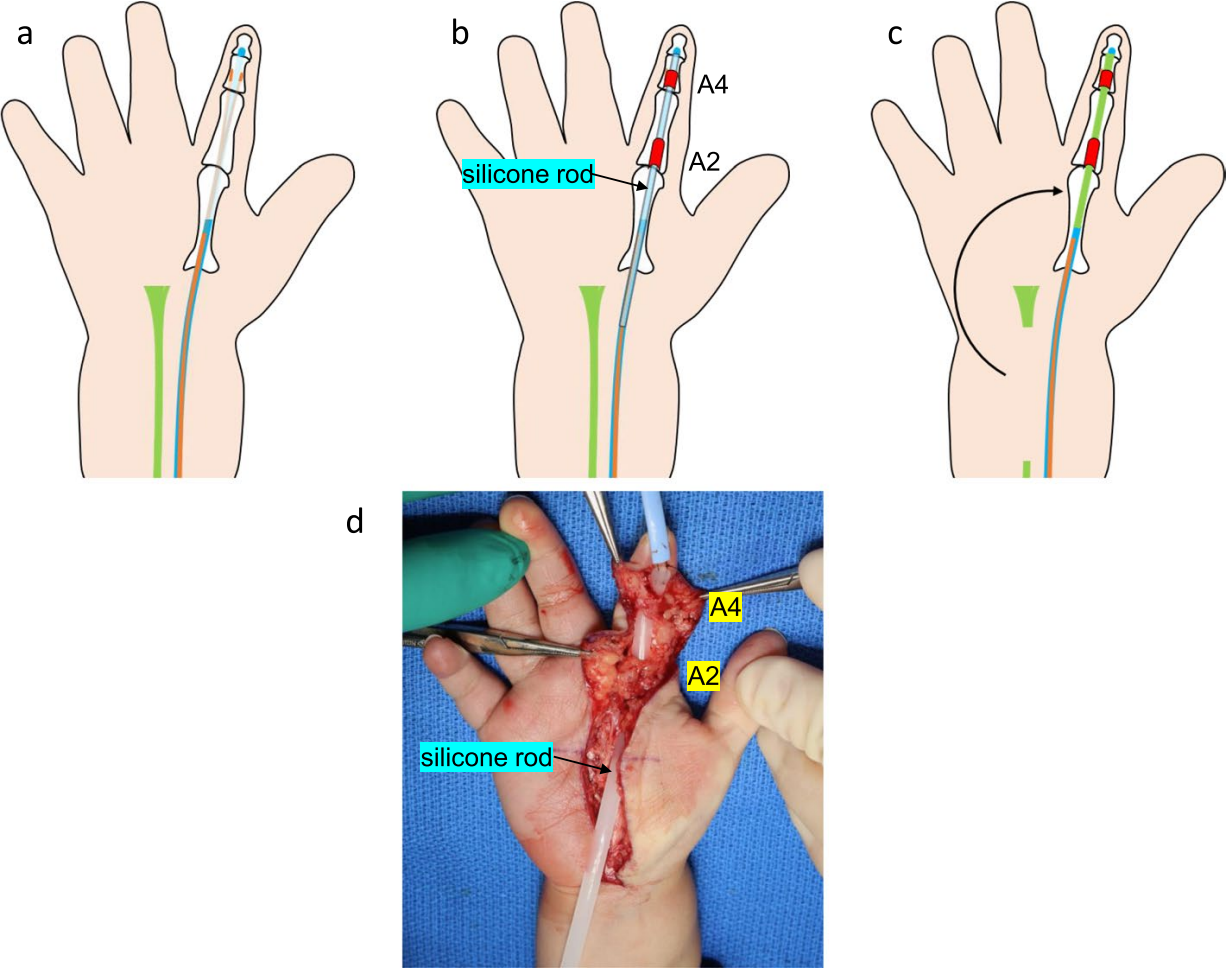
**a** The flexor digitorum superficialis (FDS) tendon (orange line) and the flexor digitorum profundus (FDP) tendon (blue line) are torn and cannot be visualized. **b** For the right index finger, a two-stage procedure was performed wherein a silicone rod (blue line) with a diameter of 3 mm was inserted, and A2 and A4 pulley reconstruction was done using the scar and remnant tendon. **c** For the second stage, the rod was replaced with the palmaris longus tendon (green line) graft. **d** Intraoperative image of silicone rod insertion through the reconstructed pulleys

The patient was discharged after 49 days after admission. However, a rupture of the left FPL tendon was found on regular ultrasound examination. Silicone rods were inserted into the right index finger and left thumb to induce the formation of a tendon sheath at 11 months. The rods were removed 1 month later because of postoperative MSSA infection. At the age of 1 year and 11 months, a silicone rod was re-inserted into the right index finger for the same purpose (Fig. 2a, b, d). At 2 years and 1 month, the right palmaris longus (PL) tendon was transplanted into the tendon sheath (Fig. 2c). Follow-up by orthopedic surgeons is ongoing.

### Additional immune function analysis

The bactericidal capacity of neutrophils and monocytes was within the normal range. Flow cytometry of lymphocytes confirmed that the number and percentage of T cells, B cells, and NK cells were likewise normal, as were the maturation of T cells and B cells. Serum CH50 levels were within the normal range at 3 months of age. There were no symptoms suggestive of a compromised immune system after this infection until 2 years of age.

## Discussion

We presented a rare case of a preterm neonate with pyogenic tenosynovitis, which led to multiple finger flexor tendon ruptures, arthritis, and osteomyelitis. Intravenous antibiotics should be administered within 24 h of the onset of symptoms because delayed treatment can worsen the prognosis, including dysfunction such as limited range of motion of finger joints and amputation [1–3]. Since there are no reports of neonates, data from adults indicate that complications occur in 38% of pyogenic tenosynovitis, including stiffness, deformity, deep infection, adhesions, persistent infection, tendon necrosis [2], and tendon rupture after infection [4, 5]. Poor prognostic factors include beta-hemolytic group A streptococci, and delayed surgical management [6]. The most common cause of pyogenic tenosynovitis is secondary to trauma including local inoculation via lacerations, puncture wounds, and bites, and is the most common route of bacterial entry [1]. Other risk factors include immunodeficiency and immunosuppression, smoking, and joint diseases, such as rheumatoid arthritis [1, 6, 7].

The pathogen involved in this case was *Staphylococcus aureus*, the most common bacteria identified in pediatric osteoarticular infections [8]. The suspected site of primary infection was the insertion site of the infusion needle. In Japan's NICU, wiping the puncture site with a packet of cotton impregnated with 80% ethanol before administering the infusion is standard practice. Preterm infants are at risk of skin infections because of the thin keratin and poor epidermal barrier [9]. The skin barrier takes 2–4 weeks to fully mature [10]. Skin sterilization before and after securing the intravenous route may have been insufficient in this case. Infections may be fatal for neonates [11]; thus, it is important to avoid infusion whenever possible and start enteral nutrition early to minimize infections from infusion needle insertion.

MSSA was detected in blood and synovial fluid cultures, and CRP was significantly elevated on admission. However, procalcitonin was minimally increased, and white blood cell counts were within the normal range for a preterm neonate. Serum PCT levels rise quickly but fall within the normal range within the first 72 h of life, after which time adult diagnostic criteria for bacterial infection can be used [12]. The PCT value was less than 2.0 ng/mL, the level indicative of severe sepsis [13]. These laboratory data suggested that, while the local infection was potent, the systemic infection was less aggressive. However, bacteremia may have resulted in multiple infection lesions and subsequently caused overt DIC. Inactivity and apnea of preterm infants are seen in severe medical conditions and are not specific to sepsis.

Congenital immunodeficiency was suspected because of severe, concurrent local infections. However, the tests for cell-mediated and humoral immune reactions yielded normal results. The hypocomplementemia on admission was also transient. Moreover, this case had no subsequent recurring infections or family history. Therefore, immune deficiency was not considered in this case. Genetic testing may be a better option if a patient has subsequent recurring infections or family history because diagnostic values of the test for determining immunodeficiency are not very sensitive.

US is an essential test for diagnosing pyogenic tenosynovitis with a sensitivity of 94% and a negative predictive value of 97% [14]. It was used to establish the diagnosis of pyogenic tenosynovitis in our case. Due to the tiny fingers of the preterm infant, the ultrasonographer used a high-frequency linear probe with a center frequency of 24 MHz for diagnosis. A hockey stick probe with a center frequency of 22 MHz was also used to observe the area in detail. The higher the ultrasonic frequency, the greater the attenuation, but the better the directivity and resolution, the more it is suitable for observing finger joints. The left FPL tendon had a chronic rupture. In such cases, US should be repeated if symptoms, such as limited range of motion, persist. US is also highly accurate in the early diagnosis of pyogenic arthritis. Floaters in the joint fluid seen in this case is a finding suggestive of infectious arthritis [15]. US is essential for the diagnosis of infected foci in soft tissue infections.

VCM, in conjunction with cefepime, cefotaxime, ceftriaxone, or gentamicin, is the recommended antimicrobial agent for neonatal soft tissue infections with systemic manifestations, such as bacteremia [16, 17]. As the infection in this case was hospital-associated, MEPM and VCM were initially selected. These antibiotics cover a wide range of gram-positive and gram-negative bacteria, including MRSA. However, after MSSA was identified, FMOX was prescribed instead. FMOX is a parenteral oxacephem antibiotic with high tissue transferability. It has a strong antibacterial effect against *Staphylococcus aureus* [18] and was thus administered as a single agent based on susceptibility. FMOX has been used in neonates and is a safe antimicrobial agent with few side effects [19].

When systemic signs, such as bacteremia or spread to adjacent structures are present, antibiotic treatment of pyogenic tenosynovitis typically takes four to six weeks. In this case, sepsis, pyogenic tenosynovitis, and pyogenic arthritis preceded the onset of the disease. Therefore, we initially planned four weeks of antibiotic treatment. However, osteomyelitis became apparent on weekly follow-up radiography during the 4^th^ week. In infants, pyogenic osteomyelitis is caused by hematogenous transfer or direct entry of bacteria into the bone marrow. Imaging findings may not appear in children until two weeks after onset [20]. Therefore, radiographic follow-up is essential for its detection. Pyogenic osteomyelitis is also treated for four to six weeks [8]. Thus, intravenous antibiotics were administered for another two weeks, followed by oral antimicrobial agents for four weeks.

Surgical repair of the right index finger started at the age of 1 year and 11 months. It was completed at the age of 2 years and 1 month due to the infection of the right index finger after silicone rod insertion. Two-stage tendon reconstructions are commonly performed in adults [21]. The range of motion of the joint has almost completely recovered, and the patient is being followed-up.

The limitations of this report include the inability to submit a culture of fluid stored in the tendon sheath due to the small puncture space. Additionally, our institution did not have the resources to confirm pathological changes.

## Conclusion

Neonatal pyogenic tenosynovitis is a highly emergent soft tissue infection. Despite the absence of immunodeficiency disease, it may be secondary to a severe bacterial infection, spread hematogenously against therapeutic intervention, leading to multiple tendon ruptures. When sepsis is suspected, close observation of skin changes and evaluation of joint motion are crucial. In case of joint or subcutaneous tissue swelling and restriction of joint motion are observed, US is useful for diagnosis. In addition, it is important to refer the patient immediately to a neonatal inpatient facility where a pediatric orthopedic surgeon can provide appropriate care to implement drainage, and subsequent treatment can be performed. Furthermore, long-term follow-up for the progression of joint lesions is important even after curing sepsis.”

## Data Availability

The dataset supporting the conclusions of this article is included in the manuscript.

## Abbreviations

US: Ultrasonography
MSSA: Methicillin-susceptible *Staphylococcus aureus*
PIP: Proximal interphalangeal
NICU: Neonatal intensive care unit
DIP: Distal interphalangeal
IP: Interphalangeal
DIC: Disseminated intravascular coagulation
FDP: Flexor digitorum profundus
FDS: Flexor digitorum superficialis
MP: Metacarpophalangeal
FPL: Flexor pollicis longus
MRI: Magnetic resonance imaging
MEPM: Meropenem
VCM: Vancomycin
FMOX: Flomoxef
CRP: C-reactive protein
PL: Palmaris longus
